# Preliminary Investigation on the Involvement of Cytoskeleton-Related Proteins, DAAM1 and PREP, in Human Testicular Disorders

**DOI:** 10.3390/ijms22158094

**Published:** 2021-07-28

**Authors:** Massimo Venditti, Davide Arcaniolo, Marco De Sio, Sergio Minucci

**Affiliations:** 1 Dipartimento di Medicina Sperimentale, Sez, Fisiologia Umana e Funzioni Biologiche Integrate “F. Bottazzi”, Università degli Studi della Campania “Luigi Vanvitelli”, Via Costantinopoli 16, 80138 Napoli, Italy; 2 Dipartimento della Donna, del Bambino e di Chirurgia Generale e Specialistica, Università degli Studi della Campania “Luigi Vanvitelli”, Via Luigi De Crecchio 2, 80138 Napoli, Italy; davide.arcaniolo@unicampania.it (D.A.); marco.desio@unicampania.it (M.D.S.)

**Keywords:** testis, cancer, classic seminoma, Leydig cell tumor, Sertoli cell-only syndrome, cytoskeleton, DAAM1, PREP, steroidogenesis

## Abstract

Herein, for the first time, the potential relationships between the cytoskeleton-associated proteins DAAM1 and PREP with different testicular disorders, such as classic seminoma (CS), Leydig cell tumor (LCT), and Sertoli cell-only syndrome (SOS), were evaluated. Six CS, two LCT, and two SOS tissue samples were obtained during inguinal exploration in patients with a suspect testis tumor based on clinical examination and ultrasonography. DAAM1 and PREP protein levels and immunofluorescent localization were analyzed. An increased DAAM1 protein level in CS and SOS as compared to non-pathological (NP) tissue was observed, while LCT showed no significant differences. Conversely, PREP protein level increased in LCT, while it decreased in CS and SOS compared to NP tissue. These results were strongly supported by the immunofluorescence staining, revealing an altered localization and signal intensity of DAAM1 and PREP in the analyzed samples, highlighting a perturbed cytoarchitecture. Interestingly, in LCT spermatogonia, a specific DAAM1 nuclear localization was found, probably due to an enhanced testosterone production, as confirmed by the increased protein levels of steroidogenic enzymes. Finally, although further studies are needed to verify the involvement of other formins and microtubule-associated proteins, this report raised the opportunity to indicate DAAM1 and PREP as new potential markers, supporting the cytoskeleton dynamics changes occurring during normal and/or pathological cell differentiation.

## 1. Introduction

In the adolescent and young adult male population (between 20 and 40 years), testicular cancers (TC) represent one of the most common solid tumors [1,2,3,4]. The incidence rate and mortality change significantly between different countries, with the highest percentages in the most industrialized world [5]. Over the last years, TC frequency in the western countries has been growing, probably due to an increased exposure to environmental pollutants [6], including many endocrine-disrupting chemicals [7,8,9]. On the other hand, many clinical and epidemiological data also evidenced the contribution of genetic factors to their susceptibility, even though the molecular mechanism(s) underlying this association is still unclear [10,11]. It is worth remembering that among the risk factors correlated to the onset of disease, there are age, family and personal history of TC, body size, cryptorchidism, congenital abnormalities, and sub-infertility or infertility [12].

TC is a heterogeneous pathology, which includes several types of cancer, such as germ cell tumors (GCT), sex cord–gonadal stromal tumors (SCGST), and secondary testicular tumors [12]. Therefore, they are classified and divided into different subgroups by the International Agency for Research in Cancer of the World Health Organization (WHO) [13]. GCTs, characterized by utmost phenotypic heterogeneity, are those with the most frequent occurrence (90–95% of cases) and with a positive course [14]. Classic seminoma (CS) is the most common GCT and may originate from developmentally blocked immature gonocytes that endure beyond fetal/perinatal life [15].

SCGST (Sertoli cell tumor, Leydig cell tumor (LCT)) are found in less than 5% of all TCs [16], while LCT accounts for 1% to 3% of TCs and is characterized by the overproduction of steroids (androgens in first place) and, consequently, by the onset of precocious puberty [17].

Growing evidence from the literature is pointing out that infertility is one of the consequences of TCs [18]; on the other hand, many other testicular non-neoplastic conditions may lead to infertility or subfertility. One of these is the Sertoli cell-only syndrome (SOS); indeed, it has been estimated that 5% to 10% of cases of men’s infertility between 20 to 40 years old could be attributable to SOS [19]. This syndrome is characterized, as the name suggests, by the presence of only Sertoli cells (SC) in the testicular seminiferous tubules, with absent (complete SOS) or very low (focal SOS) spermatogenesis [19]. The exact etiology of SOS is still unknown, even though chromosomal, genetic, endocrine, and environmental factors have been associated with the onset of SOS [19].

In this scenario, the complexity of the features of TCs and other testicular disorders is evident; thus, the knowledge of the biological mechanisms leading to a cancerous phenotype enables potential therapeutic and screening targets to be found. One of the major key events occurring during the neoplastic transformation, differentiation, and proliferation is the modification of cell shapes and their loss of polarity, determined by changes in the fundamental cytoskeletal proteome. Thus, the cytoskeleton, as well as the countless associated proteins, plays a pivotal role in this dynamic process [20].

It is worth remembering that the cytoskeleton, which consists of three major components (microtubules, microfilaments, and intermediate filaments), and its associated/regulatory proteins, is an adaptive and dynamic network in the cytoplasm, which determines the shape and the structural support for cells. Moreover, cytoskeletal dynamics is one of the key events occurring during the spermatogenic cycle, assisting germ cells (GC) in their development from a round diploid cell into a haploid highly specialized mature spermatozoa [21]. At the same time, the cytoskeleton allows SC to modify their shape depending on GC developing phase [21] and permits the proper formation and physiology of the blood–testis barrier (BTB) [22]. In our previous works, the possible role exerted by two cytoskeleton-associated proteins, the formin Dishevelled-associated activator of morphogenesis 1 (DAAM1) [23,24,25,26,27] and the enzyme prolyl endopeptidase (PREP) [27,28,29,30,31], involved in actin- [32] and microtubules- [33] based processes, respectively, was examined.

The purpose of this study was to evaluate, for the first time, DAAM1 and PREP expression and localization in CS, LCT, and SOS to elucidate potential relationships between these cytoskeleton-associated proteins and the above-mentioned testicular disorders.

## 2. Results and Discussion

Although they account for nearly 1% of all cancers in men worldwide, TCs are the most common solid tumor among the 15–40 age group [34]. Fortunately, TCs, also in metastatic form, are highly sensitive to the chemotherapeutic drug cisplatin, increasing the overall cure rate up to 90–95% [35,36]. On the other hand, both the disease per se and the used therapies may have, therefore, a reduced sperm quantity and quality, leading to sub- infertility [18,36] and then to the failure of the reproductive success and species survival. Infertility, which affects 15–20% of couples worldwide, and of which men contribute roughly 50%, may be also produced by a wide plethora of causes, including genetical, pathological, and environmental factors. One of the most peculiar is the SOS, characterized by the almost total absence of GC inside seminiferous tubules that are thereby occupied just by SC. Thus, beyond the clinical interest raised by these pathologies, developing the knowledge on the mechanism(s) characterizing TCs and SOS is of interest, with the ultimate scope to identify new molecular factors to be used as diagnostic/follow-up markers. 

Worth remembering is the prominent role played by the cytoskeleton and its dynamics in all the proliferative and differentiative cellular processes, especially during the spermatogenesis, where immature GC undergo both mitotic and meiotic divisions, followed by the spermiogenesis, allowing the production of good-quality spermatozoa. 

Therefore, to obtain more information on the relationship between the role of cytoskeleton dynamics changes and testicular disorders, we analyzed, in five non-pathological (NP), six CS, two LCT, and two SOS testicular tissue samples, the expression and localization of two cytoskeleton-associated proteins that, previously, we demonstrated to be involved in the regulation of the male germinal compartment cytoarchitecture: DAAM1 [23,26] and PREP [26,28]. DAAM1 belongs to the formin family and regulates the nucleation of unbranched actin filaments [32], while PREP is a serine protease, and it has also been associated with microtubules [33].

Tissues samples were obtained at the “Dipartimento della Donna, del Bambino e di Chirurgia Generale e Specialistica—Università Vanvitelli”, and the diagnosis was confirmed by histological evaluations.

**Figure 1:**
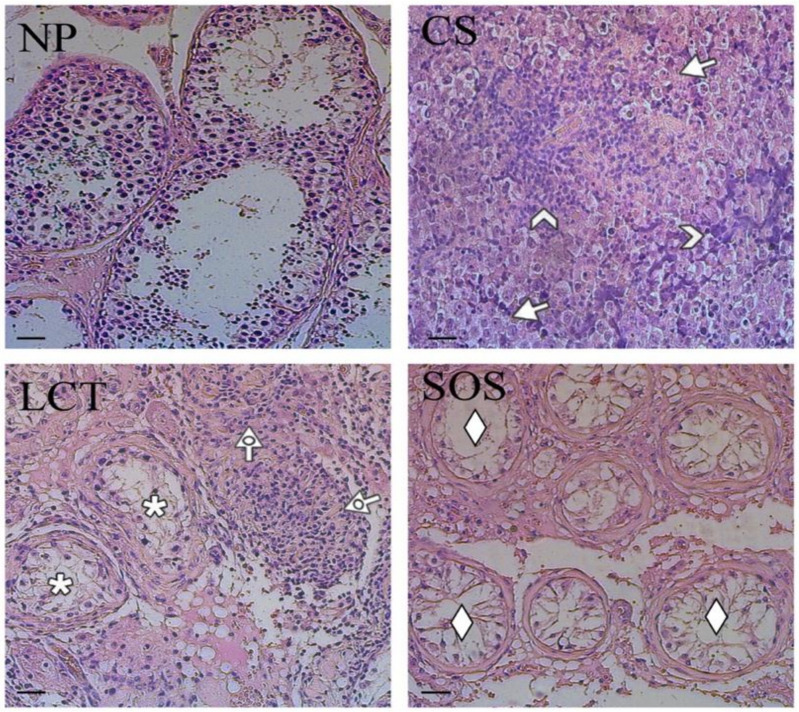
Hematoxylin-eosin staining of normal and pathological testicular tissues. Evaluation of testicular histology of non-pathological (NP), classic seminoma (CS), Leydig cell tumor (LCT), and Sertoli cell-only syndrome (SOS) samples. Arrow: seminoma cells; arrowhead: lymphocytic infiltration; dotted arrow: interstitial cells with abundant, eosinophil cytoplasm; *: testicular parenchyma; ♦: “Sertoli only” tubules. Scale bars represent 40 µm.

The representative histological features of each sample are shown in Figure 1.

The non-pathological (NP) testicular tissues were characterized by the presence of all the germ and somatic cells, exhibiting a normal spermatogenesis (Figure 1, NP). CS were characterized by the presence of unvaried dissemination of similar, rounded cells with large, centralized nuclei and nucleoli (arrows; Figure 1, CS) and by lymphocytic infiltration in the stoma (arrowhead; Figure 1, CS). In LCT, the presence of cellular, monomorphic proliferation was evident, in which the cells were characterized by abundant, eosinophil cytoplasm and a single nucleolus inside the round nucleus (dotted arrow; Figure 1, LCT). Moreover, in the periphery, testicular parenchyma was recognizable (asterisk; Figure 1, LCT). Finally, SOS samples showed testicular tissue characterized by seminiferous tubules with architectural distortion, a marked reduction of GC, and the diffused presence of “Sertoli only” tubules (rhombus; Figure 1, SOS).

**Figure 2:**
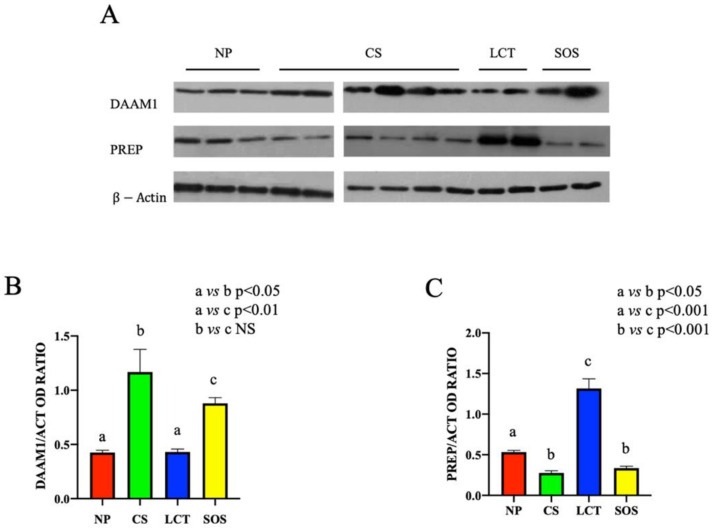
Western Blot (WB) analysis of DAAM1 and PREP in non-pathological (NP), classic seminoma (CS), Leydig cell tumor (LCT), and Sertoli cell-only syndrome (SOS) testicular tissues. (**A**): WB analysis showing the expression of DAAM1 (120 kDa); PREP (80 kDa) and β-actin (44 kDa) in testicular tissue of NP, CS, LCT, and SOS samples. (**B**,**C**): Histograms showing the relative protein levels of DAAM1 and PREP, respectively. Data were normalized with β-actin and reported as OD ratio. Values are expressed as means ± SEM.

Western blot (WB) analysis showed that the protein levels either of DAAM1 and PREP changed in the different testicular pathologic conditions (Figure 2).

Despite the variable trend of DAAM1 protein level observed in CS and SOS testicular tissues, a significant increase in each sample of the two analyzed groups, as compared to the NP group (*p* < 0.05 and *p* < 0.01, respectively), was evidenced. No significant differences in LCT samples as compared to NP group were observed (Figure 2A,B).

Conversely, regarding PREP, an opposite situation was found; in fact, its protein level strongly increased in LCT samples (*p* < 0.001), while in CS and SOS samples (*p* < 0.01), it decreased as compared to NP tissues (Figure 2A,C).

**Figure 3:**
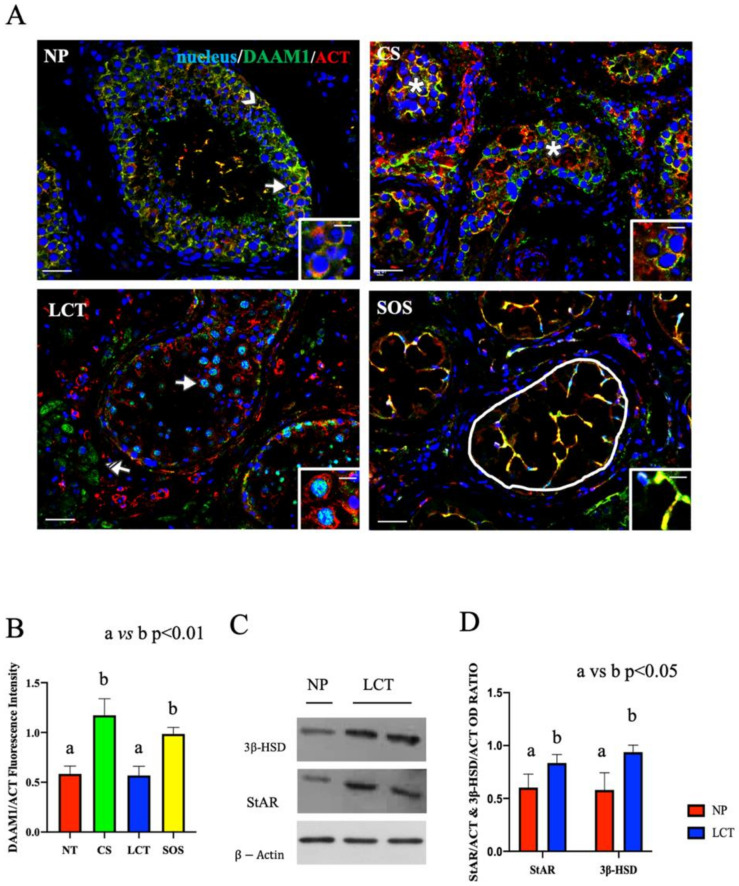
Immunofluorescence (IF) analysis of DAAM1 in non-pathological (NP), classic seminoma (CS), Leydig cell tumor (LCT), and Sertoli cell-only syndrome (SOS) testicular tissues and Western Blot (WB) analysis of steroidogenic enzymes in LCT testicular tissues. (**A**): IF analysis of DAAM1 (green) and β-actin (red) in testicular tissues of NP, CS, LCT, and SOS Scale bars represent 20 and 10 μm in the insets. Arrow: spermatogonia; arrowheads: Sertoli cells; *: seminoma cells; striped arrows: interstitial LCT cells; white round area: “Sertoli only” tubules. (**B**): Histogram showing the quantification of DAAM1 fluorescence signal intensity, respect to β-actin, using ImageJ. (**C**): WB analysis showing the expression of 3β-HSD (42 kDa); StAR (32 kDa) and β-actin (44 kDa) in testicular tissues of NP and LCT patients. (**D**): Histograms showing the relative protein levels of StAR and 3β-HSD. Data were normalized with β-actin and reported as OD ratio. Values are expressed as means ± SEM.

**Figure 4:**
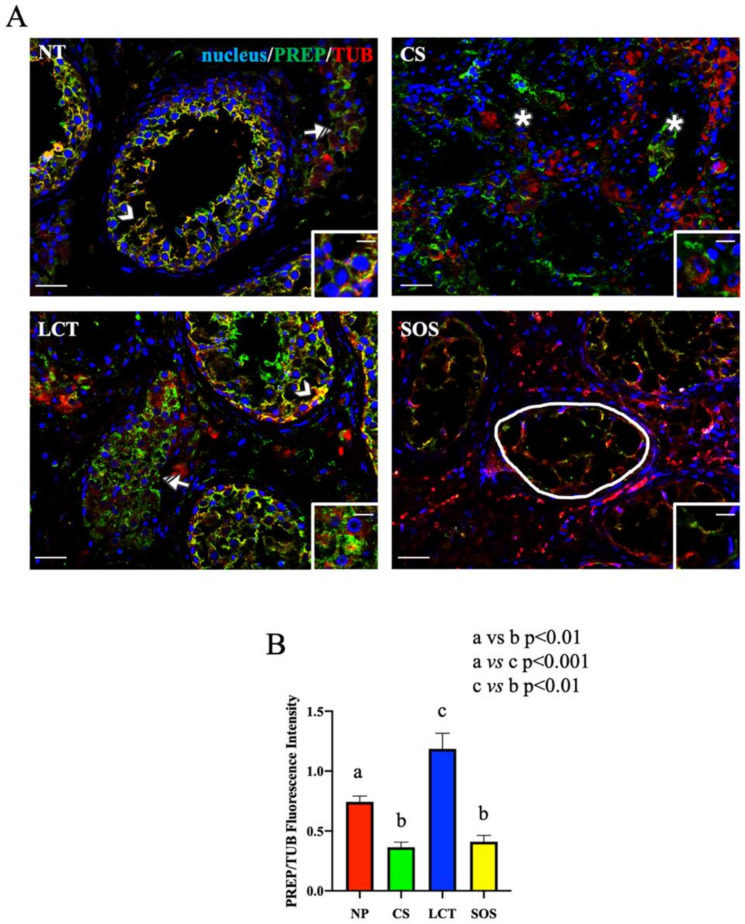
Immunofluorescence (IF) analysis of PREP in non-pathological (NP), classic seminoma (CS), Leydig cell tumor (LCT), and Sertoli cell-only syndrome (SOS) testicular tissues. (**A**): IF analysis of PREP (green) and α-tubulin (red) in testicular tissue of NP, CS, LCT, and SOS samples. Slides were counterstained with DAPI-fluorescent nuclear staining (blue) Scale bars represent 20 and 10 μm in the insets. Arrowheads: Sertoli cells; striped arrowhead: Leydig cells; *: seminoma cells; striped arrow: LCT cells; white round area: “Sertoli only” tubules. (**B**): Histogram showing the quantification of PREP fluorescence signal intensity, respect to α-tubulin, using ImageJ. Values are expressed as means ± SEM.

All the above results were strongly supported by the immunofluorescence (IF) analysis (Figure 3 and Figure 4), which revealed an altered localization and signal intensity of both DAAM1 (Figure 3) and PREP (Figure 4) in all the considered samples.

In NP samples, a specific DAAM1 localization was observed in the cytoplasm of SC (arrowhead, Figure 3A) and in that of all the GC composing the seminiferous epithelium. As expected, DAAM1 clearly co-localized with actin, its cytoskeletal partner, in the perinuclear space of spermatogonia (SPG), as highlighted by the presence of the intermediate yellow-orange tint (arrows; Figure 3A and inset).

The IF picture of DAAM1 in CS confirmed the absence of a normal seminiferous epithelium (Figure 3A). In fact, its co-localization with actin was particularly evident in the cytoplasm of round seminoma cells (asterisk; Figure 3A and inset), showing a higher fluorescent signal, as compared to the NP (*p* < 0.001; Figure 3B). It must be remembered that, in both embryonic development and in adult tissue, DAAM1, like its downstream effectors (the small GTPase RhoA and the kinase ROCK), is one of the components of the Planar Cell Polarity (PCP), a non-canonical pathway activated by Wnt ligands, which, facilitating cytoskeleton organization, regulates cell polarity, motility, and migration [37,38,39]. Additionally, in recent years, an important role for Wnt/PCP in cancer is being highlighted, since a strong correlation has been demonstrated between the up-regulation of PCP components, mainly RhoA, and the poor patient outcome in a variety of different cancer types [40,41,42,43,44,45]. Moreover, the over-expression of DAAM1 itself has been associated with the progression and invasiveness of several neoplasia, including breast [46,47,48] and prostate [49] cancers. Thus, considering this last point, and the fact that this is the first study indicating the correlation between the upregulated DAAM1 protein level and CS, it may be assumed that the involvement of this formin in the cellular events leading to neoplastic transformation. However, in this context, the role of other formins cannot to be excluded, since it has been previously demonstrated that the up-regulation of many of these family members, such as mDia, DIAPH1, and FMNL, has been positively correlated to cancer progression [50,51,52,53,54,55,56,57]. Therefore, the study of such proteins in CS may be of great interest, but it is beyond the scope of this paper, which is exclusively focused on DAAM1.

In LCT, DAAM1 localized in the cytoplasm of interstitial cells (striped arrow; Figure 3A) and, interestingly, what is worthy of note is its presence in the nucleus of the scattered SPG that were still present among the abundant tumoral Leydig cells (LC; arrow; Figure 3A and inset). Conventionally, DAAM1 has been described as a cytoplasmic protein that supports the nucleation of actin [58]; however, we recently found, for the first time, the nuclear shuttling of DAAM1 just in rat SPG, whose enhanced proliferation was induced using the excitatory amino acid D-Aspartate (D-Asp) [25]. Since D-Asp acts on the testis stimulating testosterone (T) production and SPG proliferation, we hypothesized that DAAM1 shuttling in the nucleus was needed to regulate actin dynamics in loco, as a response to the augmented cell division [25]. Here, we found a similar condition as one of the most common effects of LCT, which leads to precocious onset of puberty [59], is the enhancement of T production, highlighted also by the detected over-expression of the steroidogenic enzymes 3β-HSD (*p* < 0.05; Figure 3C,D) and StAR (*p* < 0.05; Figure 3C,D). Thus, all these combined data led us to hypothesize that, also in this case, the increased steroidogenesis, giving rise to the enhanced production and secretion of T, may consequently induce DAAM1 shuttling in the SPG nucleus to deal with cell proliferation.

Finally, DAAM1 co-localized with actin in the extensive cytoplasmic protrusions of SC, which are the only cellular elements occupying the seminiferous tubules in SOS (round area; Figure 3A,B). SC are surely the cells possessing the most dynamic cytoskeleton in the mammalian testis, needed either to “create” the BTB and also to drive developing spermatids towards the lumen [60]. This is possible due to the presence of specialized actin bundles, PCP proteins, and other regulatory factors at the basal and apical ectoplasmic specialization to support cytoskeleton function during the seminiferous cycle [22,61,62]. The described localization led us to hypothesize that the absence of the germinal component in the cell junctions may cause the wider extension of SC cytoplasm to partially compensate for the lack of BTB and to create further connections between them. Although this speculation is fascinating, it certainly needs further investigations. 

As concerns the IF results of PREP and tubulin, its cytoskeletal partner, the intermediate yellow-orange tint highlighted their cytoplasmic co-localization in most of the GC composing the seminiferous tubules, as well as their prominent localization in the cytoplasm of SC (arrowhead, Figure 4A and inset). It is interesting to note that PREP localized also in the cytoplasm of interstitial LC, but contrarily to what was found in the tubular cells, no intermediate yellow-orange tint was observed here, indicating the absence of co-localization with tubulin (striped arrow; Figure 4A). This difference may be the consequence of the two functions attributed to PREP; in fact, in the seminiferous tubular cells the association with tubulin may reflect its prominent role as a microtubule-associated protein [63], while in LC cytoplasm (where such co-localization was missing), it may act like a peptidase in controlling the hormonal homeostasis [30,64,65,66] (as further discussed later).

In CS samples, PREP specifically localized in the cytoplasm of seminoma cells (asterisk; Figure 4A and inset); however, its cellular distribution appeared disorganized, showing a less intense signal, as compared to that of NP (Figure 4B). Moreover, worthy of note is the complete absence of PREP co-localization with tubulin, which was chaotically and randomly scattered in the seminoma cells (Figure 4A). During neoplastic transformation, the reprogrammed intricate cytoskeletal network is one of the cytoplasmic factors that can support cancerous cells in the promotion of their survival, growth, and invasion [67]. Thus, the different PREP distribution, and the lack of co-localization with tubulin, accompanied by the incorrect microtubules’ organization and function, may be an index of the differentiative processes leading to neoplasia. Clearly, the involvement of other microtubules-associated proteins should be further explored in future studies, as well as to clarify whether the absent co-localization between PREP and tubulin is the cause or the consequence of microtubules disorganization.

In LCT samples, PREP specifically localized, together with tubulin, in the cytoplasm of the cells forming the seminiferous epithelium (arrowhead, Figure 4A), as well as in the tumoral interstitial cells (striped arrow; Figure 4A and inset), showing a higher fluorescent signal as compared to that of NP (*p* < 0.05; Figure 4B). As above-mentioned, beside its involvement in microtubule-associated processes, several studies reported a role for PREP in the regulation of the sex hormonal homeostasis via the cleavage of the GnRH C-terminal glycinamide residue, consequently regulating the whole hypothalamus–pituitary–testis axis [30,64,65,66]. Thus, considering that, as previously discussed, one of the most important effects of LCT is the overproduction of T, it is possible to hypothesize that the enhanced PREP protein level detected in this tumoral cells may be necessary to counteract and limit the physiological threshold levels of GnRH to compensate for the excessive secretion of androgens. Once again, our previous study using the D-Asp-treated rat model can help us to formulate this hypothesis, since we found that testicular PREP expression was enhanced, particularly in LC and just after D-Asp treatment [30], supporting a role for PREP in the regulation of the physiological hormonal levels when they are unbalanced by the presence of LCT.

Finally, in SOS samples, the PREP signal almost disappeared in the wide cytoplasmic protrusions of SC, the only cells occupying those seminiferous tubules, while tubulin staining was still evident (round area; Figure 4A,B). SC, among their numerous functions, have the role of driving the developing GC, especially spermatids, toward the lumen [52]. This transport is possible thanks to microtubules, which act as “rails” for cellular transports in the seminiferous epithelium [68,69] and also to the microtubules-associated proteins, including PREP, that regulate their dynamics. Thus, probably, the almost total absence of GC may render PREP “superfluous” in SC cytoplasm since there are no GC to be transported in the seminiferous epithelium. 

## 3. Materials and Methods

### 3.1. Tissue Samples and Hormone Evaluation

As a source of testicular tissues, biopsies were performed during inguinal exploration in patients with a suspect testis tumor based on clinical examination and ultrasonography. Biopsy was carried out from the suspect testicular lump, and the presence of cancer was confirmed by extemporaneous evaluation by an expert pathologist. The number of collected samples was 5 NP, 6 CS, 2 LCT, and 2 SOS. Each tissue sample was cut into halves: one half was quickly immersed in liquid nitrogen and stored at −80 °C for WB analysis, and another one was fixed in 10% formalin for histochemical analysis.

Before surgery, all patients underwent serum evaluation of tumor markers (alpha-fetoprotein, human chorionic gonadotropin beta, LDH) and hormonal profile (FSH, LH, Total Testosterone).

The study was conducted according to the guidelines of the Declaration of Helsinki and approved by the Ethics Committee of “Università degli Studi della Campania Luigi Vanvitelli” (protocol code 206 approved on 15 April 2019).

### 3.2. Total Protein Extraction and Western Blot Analysis

The frozen testicular tissue samples were lysed in a specific buffer (1% NP-40, 0.1% sodium dodecyl sulfate (SDS), 100 mM sodium orthovanadate, 0.5% sodium deoxycholate in phosphate-buffered saline (PBS; 13.6 mM NaCl; 2.68 mM KCl; 8.08 mM Na_2_HPO_4_; 18.4 mM KH_2_PO_4_; 0.9 mM CaCl_2_; 0.5 mM MgCl_2_; pH 7.4)) in the presence of protease inhibitors (4 mg/mL of leupeptin, aprotinin, pepstatin A, chymostatin, and phenylmethylsulfonyl fluoride). The homogenates were sonicated twice by three strokes (20 Hz for 20 s each); after centrifugation for 30 min at 10.000 g, the supernatants were stored at −80 °C [70]. A total of 40 µg was separated by 9% SDS-polyacrylamide gel electrophoresis and transferred to Hybond-P polyvinylidene fluoride membranes (Amersham Pharmacia Biotech, Buckinghamshire, UK) at 280 mA for 2.5 h at 4 °C. The filters were treated for 2 h with blocking solution (5% skim milk in Tris-buffered saline (TBS; 10 mM Tris–HCl pH 7.6, 150 mM NaCl)) containing 0.25% Tween-20 (Sigma-Aldrich Corp., Milan, Italy) before the addition of anti-DAAM1 (#HPA026605; Sigma-Aldrich Corp., Milan, Italy), anti-PREP (#ab58988; Abcam, Cambridge, UK), anti-StAR (#E-AB-15419; Elabscience Biotechnology, Wuhan, China), anti-3β-HSD (#E-AB-15112; Elabscience Biotechnology, Wuhan, China), or anti-β-Actin (#E-AB-20031; Elabscience Biotechnology, Wuhan, China) antibodies diluted 1:3.000 (for DAAM1 and PREP), 1:1000 (for StAR and 3β-HSD) and 1:5000 (for β-Actin) in the blocking solution and incubated overnight at 4 °C. After three washes in TBST (TBS including 0.25% Tween20), the filters were incubated with horseradish peroxidase-conjugated anti-rabbit immunoglobulin G (IgG; #AP307P; Sigma-Aldrich Corp., Mila, Italy) for the rabbit anti-DAAM1, anti-PREP anti-StAR, and anti-3β-HSD antibodies, or anti-mouse IgG (#AP130P; Sigma-Aldrich Corp., Mila, Italy) for the mouse anti-β-Actin, all diluted 1:10.000 in the same blocking solution. Then, the filters were washed three times again in TBST, and the immunocomplexes were revealed using the ECL-Western blot analysis detection system (Amersham Pharmacia Biotech, Buckinghamshire, UK). Signals were quantified by densitometry analysis using the software ImageJ (version 1.53 g) and adjusted relatively to β-actin levels. All the experiments were performed in triplicate.

### 3.3. Histology and Immunofluorescence Analysis

The fixed tissues were dehydrated in increasing alcohol concentrations before paraffin embedding. The 5 μm thick serial sections were stained with hematoxylin/eosin. Slides were viewed under optical microscope (Leica DM 2500, Leica Microsystems, Wetzlar, Germany), and photographs were taken using the Leica DFC320 R2 digital Camera.

For immunofluorescence staining, testicular tissue sections were permeabilized with PBS containing 0.1% Triton-X-100 for 30 min after deparaffinization and rehydration [71]. Antigen retrieval was performed by pressure cooking slides for 3 min in 0.01 M citrate buffer (pH 6.0). Later, non-specific binding sites were blocked with PBS containing 5% BSA and normal goat serum diluted 1:5. Then, sections were incubated with anti-DAAM1, anti-PREP, anti-β-actin, and anti-α-tubulin (#E-AB-20036; Elabscience Biotechnology, Wuhan, China) antibodies (all diluted 1:100 in the blocking solution) overnight at 4 °C. After two washes in TPSB (PBS containing 0.25% Tween20) and two washes in PBS, the secondary antibodies (anti-rabbit Alexa Fluor 488 (#A32731; Thermo Fisher Scientific, Waltham, MA, USA) and anti-mouse Alexa Fluor 647 (#A21236; Thermo Fisher Scientific, Waltham, MA, USA)) diluted 1:500 in the blocking mixture were added for 1 h at RT. Finally, slides were washed again, and the cells nuclei were marked with Vectashield+DAPI (H-1200-10; Vector Laboratories, Peterborough, UK). The sections were observed and captured with the optical microscope (Leica DM 5000 B + CTR 5000) with a UV lamp and saved with IM 1000 software (version 4.7.0). IF experiments have been performed in triplicate (the used images are just representative), and for the fluorescent signal analysis, 25 fields/samples, for a total or 375 NP, 450 CS, 150 LCT, and 150 SOS, have been considered and analyzed. Densitometric analysis of immunofluorescence was performed with ImageJ Software (version 1.53 g) and adjusted relatively to β-actin and α-tubulin fluorescence intensity.

### 3.4. Statistical Analysis

Data were reported as mean ± standard error (SEM). Differences between the groups were considered statistically significant at *p* < 0.05. Analyses were performed using unpaired Students’ *t*-test or one-way ANOVA; Tukey’s post hoc *t*-test was applied when appropriate with Prism 5.0, GraphPad Software (version Prism 8.4.2; San Diego, CA, USA).

## 4. Conclusions

In conclusion, this is the first report on the altered expression and localization of the cytoskeleton-associated proteins, DAAM1 and PREP, in the testicular pathologies CS, LCT, and SOS. These modifications may be the direct consequence of changes in the hormonal status, as well as by the altered seminiferous epithelium environment, evidenced by the loss of GC and, consequently, their interaction with SC, and the leakage of normal activity and connections between the two compartments. Although this study is preliminary, above all due to the reduced number of the used samples, it highlighted interesting new insight into the CS, LCT, and SOS biology, also supporting the important role of DAAM1 and PREP in the cytoskeleton dynamics changes occurring before, during, and after normal and/or pathological cell differentiation. Further studies are needed to confirm all the above results, not only to verify if other formins and microtubule-associated proteins may be involved in the testicular differentiative changes but also to raise the possibility of using DAAM1 and PREP as new potential markers to confirm the diagnosis of testicular diseases.

## Data Availability

The authors confirm that the data supporting the findings of this study are available within the article.
